# DEXA measures of body fat percentage and acute phase proteins among breast cancer survivors: a Cross-Sectional Analysis

**DOI:** 10.1186/1471-2407-12-343

**Published:** 2012-08-08

**Authors:** Anne Dee, Roberta McKean-Cowdin, Marian L Neuhouser, Cornelia Ulrich, Richard N Baumgartner, Anne McTiernan, Kathy Baumgartner, Catherine M Alfano, Rachel Ballard-Barbash, Leslie Bernstein

**Affiliations:** 1 Department of Preventive Medicine, Room 418D 2001 Soto Street, MC9239, Los Angeles, CA 90089, USA; 2 Division of Public Health Sciences, Fred Hutchinson Cancer Research Center, Seattle, WA 98109, USA; 3 Office of Cancer Survivorship, Division of Cancer Control and Population Sciences, National Cancer Institute/NIH/DHHS, Bethesda, MD, USA; 4 Applied Research Program, Division of Cancer Control and Population Sciences, National Cancer Institute, Bethesda, MD 20892, USA; 5 Department of Epidemiology and Population Health, School of Public Health and Information Science, University of Louisville, Louisville, KY 40202, USA; 6 Division of Cancer Etiology, Department of Population Sciences, Beckman Research Institute, City of Hope, Duarte, CA 91010, USA

## Abstract

**Background:**

C-reactive protein (CRP) and Serum amyloid A protein (SAA) increases with systemic inflammation and are related to worse survival for breast cancer survivors. This study examines the association between percent body fat and SAA and CRP and the potential interaction with NSAID use and weight change.

**Methods:**

Participants included 134 non-Hispanic white and Hispanic breast cancer survivors from the Health, Eating, Activity, and Lifestyle Study. Body fat percentage, measured with Dual Energy X-ray Absorptiometer (DEXA), and circulating levels of CRP and SAA were obtained 30 months after breast cancer diagnosis.

**Results:**

Circulating concentrations of CRP and SAA were associated with increased adiposity as measured by DEXA after adjustment for age at 24-months, race/ethnicity, dietary energy intake, weight change, and NSAID use. Survivors with higher body fat ≥35% had significantly higher concentrations of CRP (2.01 mg/l vs. 0.85 mg/l) and SAA (6.21 mg/l vs. 4.21 mg/l) compared to non-obese (body fat < 35%). Women who had gained more than 5% of their body weight since breast cancer diagnosis had non-statistically significant higher geometric mean levels of CRP and SAA. Mean levels of CRP and SAA were higher among obese women who were non-users of NSAIDs compared to current users; the association with SAA reached statistical significance (Mean SAA = 7.24, 95%CI 6.13-8.56 for non-NSAID; vs. 4.87; 95%CI 3.95-6.0 for NSAID users respectively).

**Conclusions:**

Breast cancer survivors with higher body fat had higher mean concentrations of CRP and SAA than women with lower body fat. Further assessment of NSAID use and weight control in reducing circulating inflammatory markers among survivors may be worthwhile to investigate in randomized intervention trials as higher inflammatory markers are associated with worse survival.

## Background

C-reactive protein (CRP) and serum amyloid A (SAA) are nonspecific acute-phase proteins that increase in response to systemic inflammation [1]. The high levels of these proteins among the obese (BMI > 30) may indicate a low-grade chronic inflammatory condition, which could result from the expansion of blood vessels and other supporting structures necessary for growth of adipose tissue [2]. Obese individuals have been shown to have higher circulating levels of pro-inflammatory cytokines (e.g. TNF-α and IL-6) and acute-phase proteins (including CRP and SAA) [3]. About one-third of circulating IL-6 comes from adipose tissue, which is also proportionally and positively associated with the over-expression of TNF-α [4,5]. The pro-inflammatory cytokine IL-6 has a dramatic impact on the secretion of acute-phase proteins by the liver and may result in a 10 to 100 fold increase in circulating CRP and SAA [6]. The inflammatory process is considered critical to both the development and progression of cancer [7,8]. Elevated circulating levels of CRP [9] and SAA [10] have been associated with greater probability of breast cancer death and with more advanced disease stage at diagnosis [11].

Previous studies evaluating the relationship between adiposity and concentrations of CRP and SAA have used anthropometric measures of obesity including body mass index (BMI, kg/m^2^), waist circumference, and bioelectrical impedance [2,12-14]. One small (N = 61) study of obese, white women found a positive association (p < 0.005) between plasma CRP and total body fat mass using Dual Energy X-ray Absorptiometry (DEXA) [15]. In our analysis, we measured body fat among breast cancer cases selected irrespective of weight. We selected DEXA as our primary measure of adiposity as it provides a highly valid and reliable estimate of total body fat in postmenopausal women, because it incorporates measures of bone mineral mass, lean soft tissue, and fat mass [16,17].

We investigated the relationship between body fat percentage and systemic inflammatory markers among a sample of Hispanic and non-Hispanic White breast cancer survivors enrolled in the HEAL (Health, Eating, Activity and Lifestyle) Study by using fat percentage assessed by DEXA and CRP/SAA measurements from samples taken at the same assessment. We explored whether the association between obesity and CRP/SAA in breast cancer survivors differs by modifiable lifestyle factors, such as weight change, or use of NSAIDs. While the biological mechanism is not known, elevated concentrations of post-diagnostic serological CRP and SAA and high post-diagnostic BMI have been associated with poor prognosis in breast cancer patients [10,18], therefore it is important to understand the factors that potentially influence these protein levels in breast cancer survivors.

## Methods

### Study population

The data for this analysis were collected for the HEAL Study, a population-based prospective cohort of breast cancer survivors which includes women who were diagnosed with in-situ to stage IIIa breast cancer from 1996 through 1999. Baseline data were collected within the first year after diagnosis, on average 7.5 months post diagnosis and follow-up data were collected approximately 24 months after baseline. The HEAL study included 1,183 women, 18 years of age or older, who were identified through the Surveillance, Epidemiology, and End Results (SEER) registries in New Mexico, Los Angeles County, California and western Washington. Of these, 615 women were recruited from New Mexico, 202 from Washington and 366 from Los Angeles. This observational study was designed to evaluate the independent roles of sex-hormones, diet, weight, physical activity, genetics, and other factors on post-diagnostic breast cancer prognosis and survival. Details of study design and recruitment procedures have been described previously [12,19,20]. The study was conducted at participating centers with the approval of respective Institutional Review Boards according to an assurance filed and approved by the U.S. Department of Health and Human Services.

**Figure 1:**
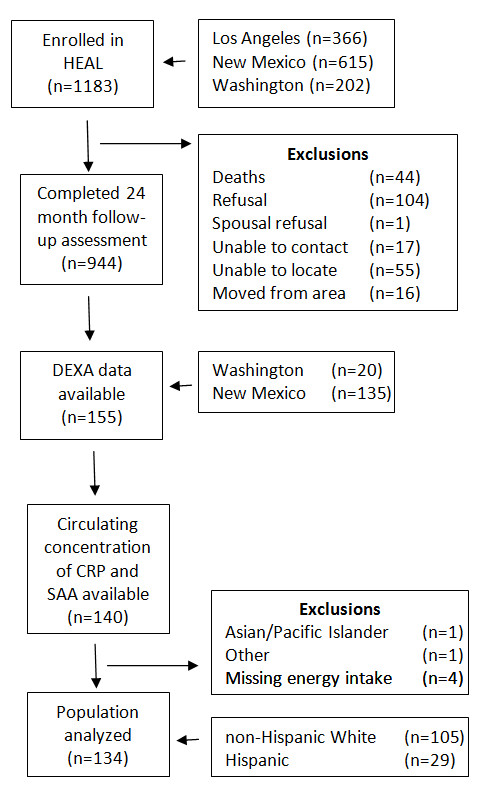
**Definition of study population and exclusion criteria.** Flow chart of the population analyzed within the HEAL cohort.

The current analysis was restricted to a sub-set of HEAL participants who had body composition measured by DEXA approximately 24 months following the initial assessment when most women had completed breast cancer treatment. Measurements of inflammatory markers (CRP/SAA) were tested at 24 months follow-up, but not at baseline. Of the 1,183 HEAL participants, 608 women completed DEXA at baseline and funds were available to conduct DEXA measurements on 155 of these patients (135 from New Mexico, 20 from Washington) at the 24 month follow-up examination. The 134 women eligible for this analysis included 17 from Washington and 117 from New Mexico who were self-identified as non-Hispanic White (n = 105) or Hispanic (n = 29). A flowchart showing reasons for exclusion is shown in Figure 1.

### Clinical variables

Trained staff at the respective study centers obtained waist and hip circumferences (in centimeters) at baseline and at the 24 month follow-up visit. Waist circumference was measured just above the superior margin of the iliac crests. Hip circumference was measured at the maximal posterior projection of the buttocks.

Body fat mass was measured using DEXA for the New Mexico and western Washington study centers at baseline and at the 24 month follow-up examinations. The Los Angeles Study center did not obtain DEXA measures. A whole body DEXA scan uses the differential attenuation of two low dose x-ray beams to partition total body mass into bone, lean and fat soft tissue components based on established mass-attenuation constants for bone mineral and lipid. Percent body fat is calculated using the bone, lean, and fat mass to estimate total fat mass divided by measured weight x 100. Measurements were taken from participants in New Mexico using the Lunar model DPX (GE Medical Systems, Milwaukee, WI) and in Washington using the Hologic model QDR 1500 (Hologic Inc, Waltham, MA). The technical error of precision for the measurement of fat mass using these devices is approximately 1.5% [21]. Furthermore, we calculated BMI using height and weight measured at the same time as the DEXA was performed at baseline and at 24 months of follow-up.

Circulating concentrations of CRP and SAA were measured by latex-enhanced nephelometry using highly sensitive assays on the Behring Nephelometer II analyzer (Dade Behring Diagnostics, Deerfield, IL) at the University of Washington. Tests were completed using fasting blood samples collected at the 24 month follow-up assessment. Each sample was processed within 3 hours of collection and stored at −70° to −80°C until analysis. Interassay coefficients of variation were 5% to 9% for CRP and 4% to 8% for SAA. The lowest detectable value for CRP is 0.2 mg/L; the lowest for SAA is 0.7 mg/L. The control materials that were included with assay batches for quality control purposes came from Bio-Rad Laboratories (Hercules, CA).

### Questionnaire variables

In-person interviews (New Mexico) and self-administered questionnaire forms (Washington) provided information on demographics, dietary intake, menopausal status, smoking status, disease history (arthritis, chronic lung disease, diabetes, heart attack, heart failure, hypertension, other cancers) and current use of any over the counter or prescription NSAIDs at the 24-month follow-up survey. Cancer treatment history, including history of radiation, chemotherapy and tamoxifen use, was obtained through medical record review, participants’ SEER records, or responses to the questionnaire. Weight gain is defined as an increase of more than 5% in body weight from the baseline to the follow-up exam and weight loss is defined as decrease of more than 5% in body weight from baseline to follow-up at 24 months [22].

Physical activity was assessed using the Modifiable Activity Questionnaire developed by Kriska and colleagues [23]. The type, duration, and frequency of activities performed in the past year were evaluated at the baseline and at the 24 month assessment. Hours of activity per week for each activity type were calculated by multiplying the frequency of each activity by the duration. Activities were further classified by intensity – light (<3 METs), moderate (3–6 METs) or vigorous (>6 METs) – based on the assignment of MET values to activities by the Compendium of Physical Activities [24]. A metabolic equivalent task (MET) is defined as the ratio of the associated metabolic rate for a specific activity divided by the resting metabolic rate (RMR). A summary measure of activity duration and intensity (restricted to moderate intensity and vigorous intensity exercise activities and defined as the sum of each activity’s MET value times hours per week) was created and used.

Energy intake was estimated using data from the Women’s Health Initiative food frequency questionnaire and the nutrient database from the University of Minnesota’s Nutrition Coordinating Center’s Nutrition Data Systems for Research (NDS-R, version 2005) [25]. Women at the New Mexico study site were asked their usual dietary intake in the preceding year, while women at the Washington site were asked about their intake in the preceding month.

### Statistical analyses

Values of CRP and SAA were logarithmically transformed due to the skewed distribution of the data. Geometric means and 95% confidence intervals (CI) were calculated for CRP and SAA concentrations by obesity status as defined by DEXA measurements (<35% versus ≥35% body fat [26]). β coefficients and 95% CIs were calculated from linear regression models to assess the associations between percent body fat and continuous values of CRP and SAA. Age, race/ethnicity, NSAID use at 24 month follow-up assessment; menopausal status at 24 month follow-up; history of chemotherapy, arthritis, hypertension, smoking, alcohol intake, energy intake at 24 month follow-up assessment; weight change between baseline and 24 month follow-up assessments; study center; physical activity at 24 month follow-up assessment, and change in physical activity between baseline and 24 month follow-up assessment were considered as potential effect modifiers and confounders in the models. All final models were adjusted for age at 24-months, race/ethnicity, dietary energy intake (kcal/day continuous), weight change (kg continuous), and NSAID use (yes/no). Other covariates were not included in the final model as they did not substantially affect our results with the exception of history of arthritis in the models for SAA. The Scheffé multiple comparison procedure (overall p < .05) was used to compare differences across groups.

Participants were classified as obese if their body fat percentage was equal to or greater than 35%, which is the standard used by most clinicians [26]. It has been described as approximately equivalent to a BMI of 25 kg/m^2^ or above in white women ages 40–59 years, however older women may have higher percent body fat associated with the same BMI [27]. Physical activity cutpoints are based on recommended activity for weight maintenance in METS (<13MET hr/wk, 13-26MET hr/wk, >26MET hr/wk) [28]. Smoking history at the 24 month follow-up assessment was classified as never smoked, smoked ≤6 months ago, and smoked >6 months ago, as we suspected that time since cessation of smoking might influence CRP or SAA levels at blood draw. Alcohol consumption at the 24 month follow-up assessment was defined as none, <10gm of alcohol, and >10gm of alcohol per day [29].

Regression analyses were used to determine the associations between CRP or SAA and BMI as well as to determine the associations between CRP or SAA and DEXA. The results for these two different anthropomorphic measures were compared. Correlations of continuous BMI values and percent body fat values measured by DEXA with log_e_(CRP) and log_e_(SAA) were examined. Statistical analyses were performed using SAS version 9.2 (SAS Institute Inc, North Carolina, USA).

## Results

Table 1 shows that breast cancer patients who were obese (i.e., body fat >35%) were more likely to be older (p < .0001), post-menopausal (p < .0001) withlarger average hip circumference (p < .0001) and larger average waist circumference (p < .0001) than women who were not obese. Despite these differences, there is an overlap in the weight ranges for the non-obese and obese (body fat >35%); the ranges were 46.0 kg - 71.0 kg and 51.0 kg-104 kg, respectively. An overlap in weight is also observed when stratifying based on BMI (obese >30 BMI) - the range for the non-obese is 17.4 kg/m^2^- 30.2 kg/m^2^ and for the obese, 20.9 kg/m^2^- 42.2 kg/m^2^.

**Table 1:** Descriptive characteristics of 134 female breast cancer survivors in HEAL Study

	**Not obese (n = 39)**	**Obese**^**a**^**(n = 95)**	**p-value**
**Characteristics at 24 month post-diagnosis assessment**			
**CRP (mg/L),***Mean (SD)*	1.2 (1.5)	3.4 (3.7)	<.0001
**SAA (mg/L),***Mean (SD)*	4.2 (2.2)	9.3 (17.9)	0.008
**Age at assessment,***Mean (SD)*	53.2 (8.8)	61.4 (10.3)	<.0001
**Postmenopausal**^b^**- yes,***n (%)*	18 (52.6)	81 (91.0)	<.0001
**Weight (kg)**^c^**,***Mean (SD)*	58.9 (7.2)	74.3 (12.1)	<.0001
**BMI (kg/m**^**2**^**),***Mean (SD)*	21.9 (2.5)	28.4 (4.3)	<.0001
**Percentage of body fat,***Mean (SD)*	29.7 (5.0)	42.6 (4.7)	<.0001
**Hip Circumference (cm),***Mean (SD)*	96.0 (5.5)	110 (9.3)	<.0001
**Waist (cm),***Mean (SD)*	74.2 (7.2)	92.4 (11.2)	<.0001
**Weight Change,***n (%)*			0.06
Loss	7 (18.0)	5 (5.3)	
No Change^d^	22 (56.4)	66 (69.5)	
Gain	10 (25.6)	24 (25.3)	
**Physical Activity (MET hrs/week),***n (%)*	44.7 (45.4)	28.2 (24.9)	0.04
**Change in Physical Activity since baseline**^e^**(MET-hrs/week)**	6.7 (29.5)	-5.3 (31.6)	0.04
**History of Arthritis** – yes, *n (%)*	5 (12.8)	50 (52.6)	<.0001
**Hypertension**^f^	6 (15.4)	31 (33.0)	0.04
**Past and current medication use - yes,***n (%)*			
Tamoxifen^g^	14 (35.9)	41 (43.2)	0.44
NSAID	8 (20.5)	40 (42.1)	0.02
Chemotherapy^h^	9 (23.1)	19 (20.0)	0.69

^a^ Obese ≥35% Body Fat, not obese < 35% Body Fat.

^b^ I non obese and 6 obese excluded due to unknown menopausal status.

^c^ Weight at follow-up is taken at the second DEXA measurement.

^d^ Weight change within 5% change in body weight since baseline.

^e^ Baseline is approximately 6 months post-diagnosis. Follow-up is approximately 30 months post-diagnosis.

^f^ 1 obese participant answered "Don't know" and was excluded.

^g^ Current use of tamoxifen or over the counter or prescription non-steroidal anti-inflammatory drug (NSAID).

^h^ 2 non-obese participants refused to answer and were excluded.

History of arthritis (p < .0001) and hypertension (p = 0.04) were significantly associated with obesity but there were no differences in several other conditions (i.e. chronic lung disease, diabetes, heart attack or heart failure, and hypertension) that were not common in the study population (data not shown). Obesity status was not associated with demographic or lifestyle factors including alcohol consumption, smoking history, or education level (data not shown) and, the proportions of obese and non-obese survivors who gained 5% or more of their bodyweight over the two year follow-up period were similar (25.3% vs. 25.6%). Neither tamoxifen therapy nor past treatment with chemotherapy was associated with obesity status. However, obese women were more likely to report current use of any prescription or over the counter NSAID at the 24 month follow-up assessment (p = .02).

Geometric mean CRP concentrations were significantly higher among obese than non-obese women (Table 2). In this table, stratum specific geometric means labeled with different numbers (1–3) are statistically significantly different from one another based on Scheffé multiple comparison procedures; values for CRP and SAA were tested separately. Obese women had higher CRP values than non-obese women among those who did not use NSAIDS compared to those who used NSAIDS. In the same way, women who gained weight from baseline to the 24-month follow-up assessment had higher mean levels of CRP than women who did not gain weight; however, these differences did not reach statistical significance. No significant differences were observed by smoking history, physical activity, or caloric intake (data not shown). We observed a dose–response relationship between CRP and tertile of percent body fat in all women (i.e. not obese and obese) combined (p < .001, data not shown). The significant positive trend persisted after adjustment for NSAID use, level of caloric intake, and history of arthritis (data not shown).

**Table 2:** Adjusted geometric means and 95% CI of CRP and SAA stratified by obesity status at 24-month follow-up assessment (n = 134)

**Stratifying variable**^a^	**CRP Mean (95%CI)**		**SAA Mean (95%CI)**	
	**Not obese (N = 39)**	**Obese**^**b**^**(N = 95)**	**Not obese (N = 39)**	**Obese (N = 95)**
**Overall**	0.85 (0.61-1.20)^1^	2.01 (1.64-2.46)^2^	4.21 (3.34-5.30)^1^	6.21 (5.42-7.11)^2^
**NSAID use**^c^				
No (n = 31/55)^d^	0.84 (0.58-1.21)^1^	2.31 (1.78-3.00)^2^	4.29 (3.36-5.48)^1^	7.24 (6.13-8.56)^2^
Yes (n = 8/40)	0.99 (0.49-1.97)^1,2^	1.64 (1.20-2.23)^1,2^	4.59 (2.93-7.19)^1,2^	4.87 (3.95-6.00)^1^
**History of arthritis**				
No (n = 34/45)	0.81 (0.57-1.17)^1^	1.89 (1.41-2.52)^2^	3.70 (2.93-4.67)^1^	5.37 (4.46-6.47)^1,2^
Yes (n = 5/50)	0.91 (0.38-2.18)^1,2^	2.19 (1.65-2.90)^2^	4.06 (2.31-7.16)^1,2^	7.75 (6.46-9.31)^2^
**Weight Change**^e^				
Loss (n = 7/5)	0.75 (0.36-1.57)^1,2^	1.96 (0.81-4.78)^1,2^	3.11 (1.94-4.98)^1^	5.27 (2.97-9.35)^1,2^
No Change (n = 22/66)	0.70 (0.46-1.07)^1^	1.93 (1.51-2.47)^2^	4.28 (3.23-5.67)^1,2^	5.88 (4.99-6.92)^1,2^
Gain (n = 10/24)	1.12 (0.59-2.12)^1,2^	2.50 (1.69-3.69)^2^	4.51 (2.97-6.86)^1,2^	7.81 (6.07-10.04)^2^

^a^ Scheffé multiple comparison procedure (p < .05) was used to compare differences across groups. For each variable means with different numbers (1-3) are statistically different form one another and evaluated separately for CRP and SAA.

CRP means are adjusted for age, race/ethnicity, energy (kcal), NSAID (yes/no), and weight difference (kg), SAA means are additionally adjusted for arthritis (yes/no).

^b^ Obese ≥35% Body Fat, not obese < 35% Body Fat.

^c^ Current use of over-the-counter or prescription non-steroidal ant-inflammatory drugs (NSAID). Adjusted for age, ethnicity, energy (kcal), weight difference (kg).

^d^ Sample size (n) represents number of non-obese and obese women in each stratum. e.g. 31 non-obese and 35 obese women who are non-users of NSAID. The same formatting in describing sample size is used for other strata.

^e^ Adjusted for age, ethnicity, energy (kcal), NSAID (yes/no). Weight gain is >5% increase in bodyweight since baseline and weight loss is >5% decrease since baseline.

Similar to our findings for CRP, geometric mean levels of SAA were significantly higher for obese than non obese participants (Table 2). Further, mean SAA concentrations were significantly higher among obese women not taking NSAIDs compared to women taking them. Higher geometric mean concentrations of SAA also were found for women who gained weight from baseline to the 24-month assessment, but these associations did not reach statistical significance. These associations were not significantly altered in a model that additionally adjusted for study site (data not shown). The associations observed were similar when using an obesity cut-point of ≥ 41% body fat as proposed by Gallgather et al. (data not shown) [27].

**Figure 2:**
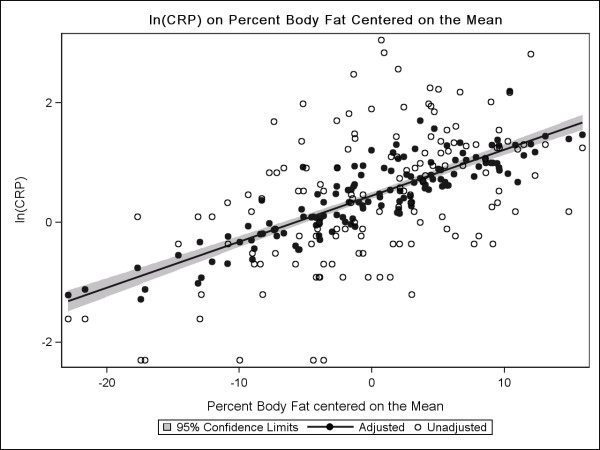
**Scatterplot and predicted regression line of log**_**e**_**(CRP) centered on body fat percentage.** Scatterplot and predicted regression line of log_e_(CRP) centered on body fat percentage on a sample of 134 breast cancer survivors from the HEAL study controlling for age, caloric intake (kcal), weight difference(kg), and NSAID use.

Figure 2 presents the plot for log_e_ (CRP) versus percent body fat centered on the mean. We observed an approximate linear relationship between log_e_ (CRP) and percent body fat, both in an unadjusted model and after adjusting for age, energy intake, weight difference, race/ethnicity and NSAID use. Our linear regression model indicates that the estimated CRP increases by 1.07 mg/L for each 1% increase in body fat beyond the mean. At the mean percent body fat of 38.9%, the estimated CRP based on the adjusted model is 1.6 mg/L. The slopes (βs) for the unadjusted and adjusted models were similar.

**Figure 3:**
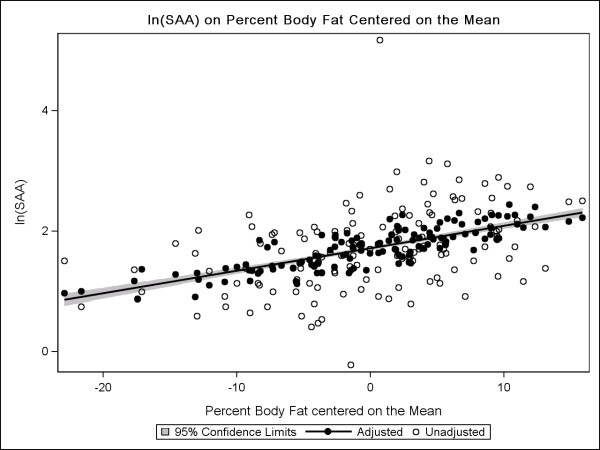
**Scatterplot and predicted regression lines of log**_**e**_**(SAA) centered on body fat percentage.** Scatterplot and predicted regression lines of log_e_(SAA) centered on body fat percentage on a sample of 134 breast cancer survivors from the HEAL study controlling for age, history of arthritis, caloric intake (kcal), weight difference(kg), and NSAID use.

Figure 3 presents the plot for log_e_ (SAA) versus percent body fat centered on the mean; we found that for every 1% increase in body fat above the mean, estimated SAA increased 1.03 mg/L. At the mean body fat percentage of 38.9%, the estimated SAA is 5.6 mg/L.

Table 3 describes the β coefficients and 95% confidence intervals (CIs) for the regression models describing the relationship between percent body fat and log_e_ (CRP) and log_e_ (SAA). The β coefficients represent the increase in log_e_ (CRP) or log_e_ (SAA) per 1% increase in body fat. The values for the overall model and those stratified by NSAID use, history of arthritis, and weight change are shown. No significant interaction was found between percent body fat and NSAIDS, history of arthritis, weight change (Table 3), or any lifestyle or health-related factor. When we stratified by recent NSAID use, we found a greater increase in log_e_ (CRP) for each 1% increase in body fat for women who did not take NSAIDs compared with women who did. The results for log_e_ (SAA) are generally similar to those described for log_e_ (CRP), although the differences in β coefficients are smaller between the strata.

**Table 3:** **Linear regression models for log **_**e **_**(CRP) and log **_**e **_**(SAA) on body fat percentage overall and stratified (n = 134) **

	**log**_**e**_**(CRP)**				**log**_**e**_**(SAA)**			
	**β**	**SE**	**95%CI**	**p interaction**	**β**	**SE**	**95%CI**	**p-interaction**
**Full Model**^**a**^	0.068	0.012	(0.04,0.09)		0.032	0.008	(0.02,0.05)	
**NSAID use (n = 48)**^**b**^	0.037	0.027	(-0.02,0.09)	0.13	-0.005	0.023	(-0.05,0.04)	0.16
**No NSAID use (n = 86)**	0.076	0.013	(0.05,0.10)		0.032	0.008	(0.02,0.05)	
**History of arthritis (n = 55)**	0.057	0.024	(0.01,0.11)	0.08	0.037	0.018	(0.00,0.07)	0.45
**No History of arthritis (n = 79)**	0.069	0.015	(0.04,0.10)		0.02	0.009	(0.00,0.04)	
**Weight Change**^**c**^				0.53				0.53
**Loss (n = 12)**	0.128	0.229	(-0.43,0.69)		-0.029	0.065	(-0.19,0.13)	
**Same (n = 88)**	0.079	0.013	(0.05,0.10)		0.034	0.008	(0.02,0.05)	
**Gain (n = 34)**	0.048	0.026	(-0.01,0.10)		-0.009	0.023	(-0.06,0.04)	

^a^ log_e_(CRP) model is adjusted for age, ethnicity, energy (kcal), NSAID (yes/no), and weight difference(kg). log_e_(SAA) model is additionally adjusted for arthritis (yes/no).

^b^ Current use of over-the-counter or prescription non-steroidal ant-inflammatory drugs (NSAID). Adjusted for age, race/ethnicity, energy (kg) weight difference (kg).

^c^ Adjusted for age, energy (kcal), NSAID (yes/no). Weight gain is >5% increase in bodyweight since baseline and weight loss is >5% decrease since baseline.

We examined the distribution of acute phase proteins (log_e_ CRP, log_e_ SAA) by continuous measures of DEXA (% body fat) or BMI (data not shown) all measured at the same follow-up visit. The distributions for percent body fat and BMI were similar, however we observed slightly less clustering of data points for DEXA measures than for BMI, when plotted against the two acute phase proteins. The correlation coefficients for log_e_ (CRP) with DEXA were 0.52 (0.39-0.63) compared to 0.47 (0.34-0.60) for BMI. Similarly, the correlation coefficient for log_e_ (SAA) were 0.42 (0.27-0.54) for DEXA compared to 0.38 (0.22-0.42) for BMI.

## Discussion

In this cohort of breast cancer survivors, we found a significant association between acute phase proteins (CRP or SAA) and adiposity as measured by DEXA. Women with higher measures of adiposity had higher CRP and SAA concentrations compared to women with lower adiposity regardless of lifestyle or medical factors. Our analysis indicates that percent body fat as measured by DEXA is a strong predictor of CRP and SAA levels. Furthermore, our data suggest recent use of NSAIDS modifies the observed association of adiposity with acute phase proteins. We found that obese women who recently used NSAIDs had significantly lower geometric mean concentrations of SAA than women who did not use NSAIDS. A similar pattern was found for CRP, although the association did not reach statistical significance. The potential modifying effects of weight change did not reach statistical significance for SAA or CRP, perhaps because small numbers of women in some of the strata limited statistical power.

DEXA may provide a more accurate measure of body fat for studies of acute phase proteins than other anthropometric measures because DEXA can distinguish between bone, muscle and fat mass [30]. The accuracy of BMI as a measure of percent body fat may vary by age, gender, race or ethnicity and physical conditioning. It is an overall measure of body mass and does not provide measures of body composition. Thus, there is no absolute cross-tabulation for the DEXA definition of fat by standard BMI. Body fat determined through clinical measures of bio-impedance also may be less reliable than DEXA, as the measure tends to underestimate adiposity [16,17]. Additionally, while BMI, waist-hip ratio, and waist circumference have been used as indicators of body fatness, these measurements were found to be more closely correlated with each other than with body fatness measured by DEXA based on data from a nationally representative US population sample (National Health and Nutrition Examination Survey) [16]. In our HEAL sample, we were not able to distinguish differences in correlation between acute phase proteins (i.e. CRP or SAA) and DEXA or BMI, possibly due to the small sample.

The association between acute phase proteins and adiposity we found is consistent with earlier reports based on anthropometric measures that found increased adiposity was significantly correlated with elevated levels of CRP and SAA among breast cancer patients [14,31]. A previous study of HEAL participants (n = 741) using anthropometric measures (BMI, waist circumference) found significant associations between BMI and inflammatory proteins [14]. The association of percent body fat with levels of acute phase proteins suggests that circulating inflammatory markers are associated with greater adiposity and not just greater weight.

We also found a suggestive association between weight gain and circulating acute phase proteins in our sample. If this association is causal, weight control or weight loss may be one mechanism for controlling elevated levels of circulating inflammatory proteins in breast cancer survivors. However, one concern related to unmonitored weight loss in breast cancer survivors is sarcopenia [32], and the potential impact of concomitant fat and muscle loss on survival. In HEAL participants, sarcopenic women were approximately 3 times as likely to die from any cause and 2 times as likely to die from breast cancer as women without sarcopenia [33]. Therefore, any efforts to maintain or lose weight would need to focus on retaining lean body mass while maintaining or losing body fat [32,34].

In the literature, weight loss and physical activity have inconsistently been associated with circulating acute phase proteins. A study of obese individuals found that weight reduction following caloric-restriction was associated with reduced plasma CRP levels [15]. One study found that weight loss was associated with a significant reduction in IL-6 levels in both plasma and adipose; the authors also found a non-statistically significant reduction in CRP [35]. Bochud et al. suggested that the lack of statistical significance with CRP in this study may be due to the short duration of weight loss and the overall small amount of weight lost [31]. However, a subsequent intervention study involving 40 overweight breast cancer survivors showed no association between weight loss and CRP [36]. With respect to physical activity, it has been indicated that aerobic exercise may be more effective than flexibility or resistance training for CRP reduction [37]. The absence of differences in effect by physical activity in the current study may be due to our small sample or that the type or level of exercise was not adequately variable to produce a measurable effect. A large proportion of HEAL participants reported high levels of physical activity, such that even the obese participants reported an average of 28.5 MET-hours per week of activity.

Our data suggest that use of NSAIDs may be one way to control circulating levels of CRP/SAA in breast cancer survivors. Additional data from randomized intervention trials are needed to confirm this potential benefit. One concern is that a meta-analysis of the available evidence on cardiovascular safety of NSAIDs found that use of some types of NSAIDs were associated with elevated risk of myocardial infarction, stroke, and cardiovascular death [38]. However, cardiovascular risk varies by type of NSAID [38], which in some cases may be protective (e.g. aspirin). An analysis of data from the Nurses' Health Study (n = 4,164) found that aspirin use is associated with decreased risk of breast cancer specific death and death from any cause in breast cancer patients [39]. A meta-analysis also showed daily use of aspirin reduced incidence and metastasis of colorectal cancers and several other cancers including breast cancer [40,41]. Thus, further assessment of the association between NSAID such as aspirin and acute phase proteins in breast cancer survivors may be of value, as lower levels of CRP have been linked with longer survival and NSAIDs have been suggested as an adjuvant treatment for breast cancer [42].

A strength of this study is the use of DEXA measurement as a precise estimate of adiposity. Our study includes both non-Hispanic white and Hispanic breast cancer survivors. Differences in percent body fat and CRP or SAA levels by race/ethnicity may be expected due to previous studies that have indicated fat-patterning differs by race and ethnicity. Specifically, higher measures of central adiposity have been found in Hispanic women [43]. Since higher CRP and SAA levels adversely affect survival among breast cancer survivors, using a more precise measure of body fat (a predictor of CRP and SAA) may be useful for accurately identifying those women who could improve prognosis by decreasing body fat [10].

The primary limitation of this study is that despite being the largest study to date with comprehensive measures of body composition, BMI and inflammation, it remains a relatively small study. Therefore, a lack of difference in stratified analyses to examine the effects of various lifestyle factors and medical conditions must be interpreted with caution as statistical power is limited. The wide variance in weight and adiposity measures also is a limitation. Other limitations in our methods that may make associations more difficult to detect include differences in DEXA equipment used in Seattle and New Mexico and use of different methods of data collection (in person vs. mailed questionnaire) to collect demographic, lifestyle and medical history information. However, the impact of study center was evaluated as a covariate in all models and women from a range of BMI and percent body fat levels were measured at each center.

## Conclusion

This analysis suggests that circulating CRP and SAA levels are positively associated with percent body fat and not just greater weight. Beyond a reduction in adiposity, changes in other modifiable factors, including weight control, or use of NSAIDs, may help to decrease levels of acute-phase inflammatory proteins. However, the potential benefits or risks of NSAIDs or weight control will require further evaluation and possible exploration in a randomized clinical trial. Assessment of the association between use of NSAIDs and survival time may be worthwhile as lower levels of CRP have been linked with longer breast cancer survival and NSAIDs have been suggested as an adjuvant treatment for breast cancer [42].

## Competing interests

The author(s) declare that they have no competing interests.

## Authors' contributions

All authors contributed to the conception and design of this study and participated in the drafting of the manuscript. AD and RMC performed the statistical analyses. All authors read and approved the final manuscript.

## Pre-publication history

The pre-publication history for this paper can be accessed here:

http://www.biomedcentral.com/1471-2407/12/343/prepub
